# Phytoplankton blooms during austral summer in the Ross Sea, Antarctica: Driving factors and trophic implications

**DOI:** 10.1371/journal.pone.0176033

**Published:** 2017-04-21

**Authors:** Olga Mangoni, Vincenzo Saggiomo, Francesco Bolinesi, Francesca Margiotta, Giorgio Budillon, Yuri Cotroneo, Cristina Misic, Paola Rivaro, Maria Saggiomo

**Affiliations:** 1 Dipartimento di Biologia, Università degli Studi di Napoli Federico II, Naples, Italy; 2 Stazione Zoologica Anton Dohrn, Naples, Italy; 3 Dipartimento di Scienze e Tecnologie, Università degli Studi di Napoli Parthenope, Naples, Italy; 4 Dipartimento di Scienze della Terra, dell’Ambiente e della Vita, Università degli Studi di Genova, Genoa, Italy; 5 Dipartimento di Chimica e Chimica Industriale Università degli Studi di Genova, Genoa, Italy; University of Shiga Prefecture, JAPAN

## Abstract

During the austral summer of 2014, an oceanographic cruise was conducted in the Ross Sea in the framework of the RoME (Ross Sea Mesoscale Experiment) Project. Forty-three hydrological stations were sampled within three different areas: the northern Ross Sea (RoME 1), Terra Nova Bay (RoME 2), and the southern Ross Sea (RoME 3). The ecological and photophysiological characteristics of the phytoplankton were investigated (i.e., size structure, functional groups, PSII maximum quantum efficiency, photoprotective pigments), as related to hydrographic and chemical features. The aim was to identify the mechanisms that modulate phytoplankton blooms, and consequently, the fate of organic materials produced by the blooms. The observed biomass standing stocks were very high (e.g., integrated chlorophyll-a up to 371 mg m^-2^ in the top 100 m). Large differences in phytoplankton community composition, relative contribution of functional groups and photosynthetic parameters were observed among the three subsystems. The diatoms (in different physiological status) were the dominant taxa in RoME 1 and RoME 3; in RoME 1, a post-bloom phase was identified, whereas in RoME 3, an active phytoplankton bloom occurred. In RoME 2, diatoms co-occurred with *Phaeocystis antarctica*, but were vertically segregated by the upper mixed layer, with senescent diatoms dominating in the upper layer, and *P*. *antarctica* blooming in the deeper layer. The dominance of the phytoplankton micro-fraction over the whole area and the high Chl-a suggested the prevalence of non-grazed large cells, independent of the distribution of the two functional groups. These data emphasise the occurrence of significant temporal changes in the phytoplankton biomass in the Ross Sea during austral summer. The mechanisms that drive such changes and the fate of the carbon production are probably related to the variations in the limiting factors induced by the concurrent hydrological modifications to the Ross Sea, and they remain to be fully clarified. The comparison of conditions observed during summer 2014 and those reported for previous years reveal considerably different ecological assets that might be the result of current climate change. This suggests that further changes can be expected in the future, even at larger oceanic scales.

## Introduction

Global temperatures have risen by >1°C over the last few decades [1], and more than 75% of the heat excess has been stored in the Southern Ocean [2]. Investigations on climate change are focused intensely on this area due to a combination of biological and physical processes, although the magnitude and direction of the changes differ on a regional scale [3, 4].

These spatially heterogeneous changes will have differential effects on phytoplankton composition, productivity, and carbon sequestration, through alterations to ambient temperature, total irradiance, wavelength structure, nutrient availability, and trophodynamics [5–7]. However, our understanding of the environmental controls on phytoplankton growth and standing stocks is still incomplete, and to date, current variations that affect the pelagic food web remain mostly unknown [8, 9]. Therefore, these topics are included in the list of the 80 priority scientific questions for future Antarctic research, as identified by the 1^st^ Scientific Committee on Antarctic Research, Antarctic and Southern Ocean Science Horizon Scan [10, 11].

The Ross Sea is the most productive sector of the Southern Ocean, and thus it has a strong impact on marine biogeochemical cycles and air-sea heat and CO_2_ fluxes on a global scale [5] [12, 13]. The phytoplankton of the Ross Sea are alternatively dominated by two functional groups, diatoms and Haptophytes (e.g., *Phaeocystis antarctica*), which are typically separated both in space and time [14, 15]. The relative abundances of diatoms and *P*. *antarctica* have key roles in shaping the food web, and can impair the absorption and export of carbon to the bottom of the Ross Sea [16–19]. Diatoms usually comprise 90% of the phytoplankton along the western continental shelf and in the Terra Nova Bay polynya, where the stratification of the melting ice of the surface waters results in relatively shallow mixed layers (≤20 m) [20, 21]. In contrast, in December, *P*. *antarctica* in mucilaginous colonies comprises 95% of the phytoplankton in the Ross Sea polynya in the deep mixed layer (40–60 m). During this time frame, the colonies are largely ungrazed and exported to the deep ocean [17, 22]. In summer, *P*. *antarctica* abundance is low, and it can occur in colonies or as single cells; the latter form can directly enter the microbial food web [23, 24]. However, the spatial and temporal mosaic of phytoplankton dynamics in the Ross Sea is more complex, as there are significant inter-annual variations [25–27]. Nevertheless, *P*. *antarctica*- and diatom-dominated waters derive from a combination of multiple physical-chemical factors, which include macro-nutrients, micro-nutrients and CO_2_ concentrations, as well as the sea surface temperature [28–32]. Among these, the timing and supply of Fe input can affect the phytoplankton composition, due to the potentially distinct Fe requirements of *P*. *antarctica* and diatoms [33–36].

At the community level, the maximum quantum yield (F_v_/F_m_) and electron transfer rate of photosystem II (PSII) in photosynthesis are widely used as indicators to assess responses of phytoplankton to different levels of environmental stressors, including deficiencies in macro-nutrients and micro-nutrients [37–39]. In this regard, it is commonly stated that a decline in F_v_/F_m_ indicates a compromised photosynthetic performance [40]. Thus, understanding the combined effects of such complex changes on phytoplankton photophysiology, and unveiling the mechanisms responsible for the distribution of these two different functional groups is crucial, due to the particular biogeochemical and ecological roles that they have within Antarctic marine ecosystems [41, 42].

To shed light on the mechanisms that modulate the distribution of phytoplankton (i.e., in terms of biomass, size classes, functional groups) and their photophysiology, this study investigated three different areas of the Ross Sea during the austral summer of 2014. The phytoplankton community structure, the factors driving the blooms and the trophic implications are discussed here, as related to water mass properties and dynamics of the surface water layer (0–200 m).

## Materials and methods

### Ethics statement

All samples were obtained during the project “Ross Sea Mesoscale Experiment” (RoME) in the framework of the Italian National Antarctic Program (PNRA, 2013/AN2.04) coordinated by the Ministry of Education, University and Research (MIUR). The permission to collect samples was authorised by the National Environmental Officer of the National Agency for New Technologies, Energy and the Sustainable Economic Development (ENEA) on the base of Environmental Evaluation (Impact) Assessment of the RoME Project, following the “Protocol on Environmental Protection of the Antarctic Treaty”, Annex II, art.3.

### Study areas and sampling strategy

A multidisciplinary oceanographic cruise in the Ross Sea was conducted on the *R/V Italica* from 16 January to 3 February, 2014, in the framework of the project entitled “Ross Sea Mesoscale Experiment” (RoME).

**Fig 1:**
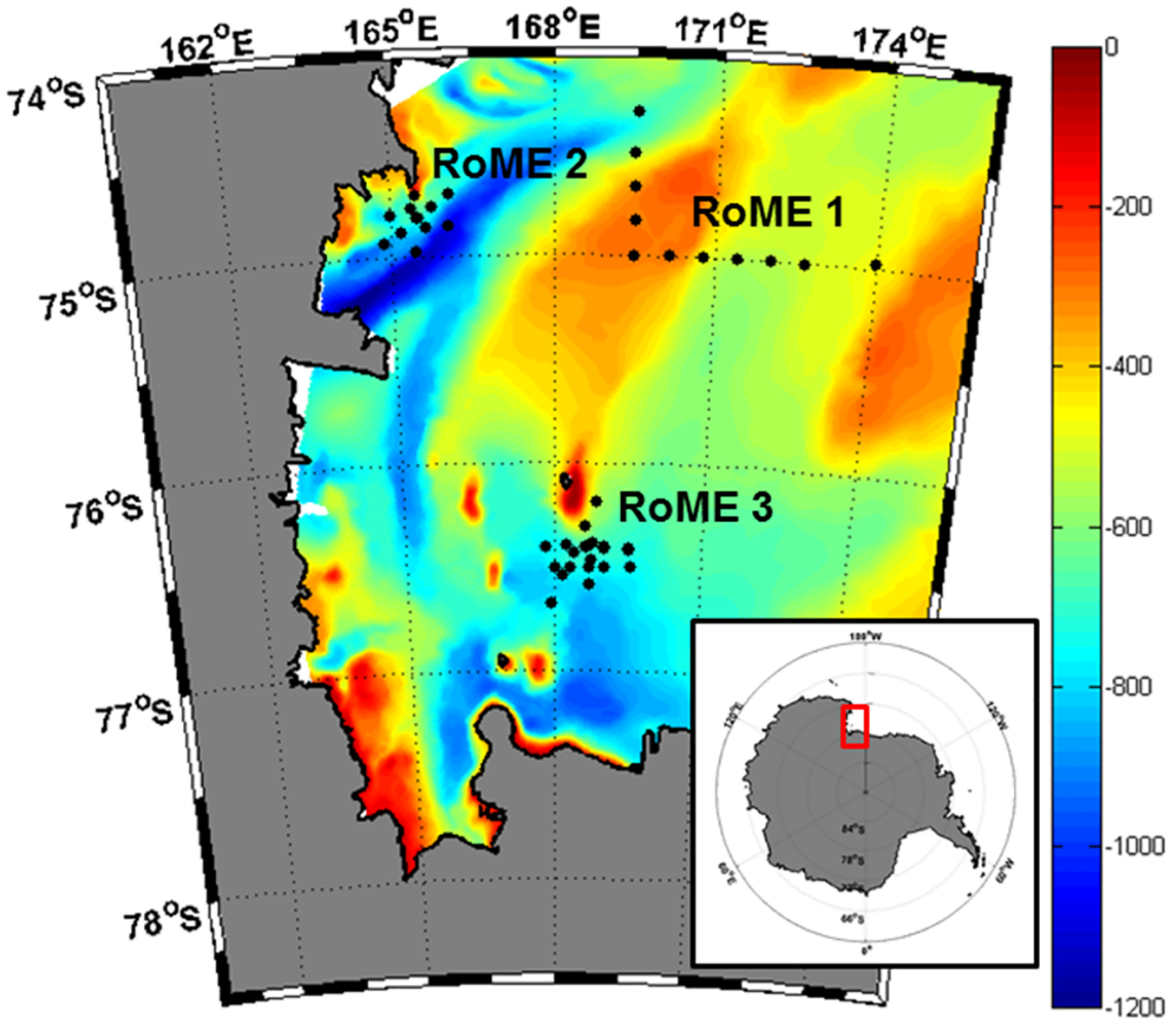
Sampling stations of the RoME Project. The black dots indicate the sampling stations. The bathymetry data of the Ross Sea from [43] under a CC BY license, with permission from [Davey], original copyright [2004–2005].

**Fig 2:**
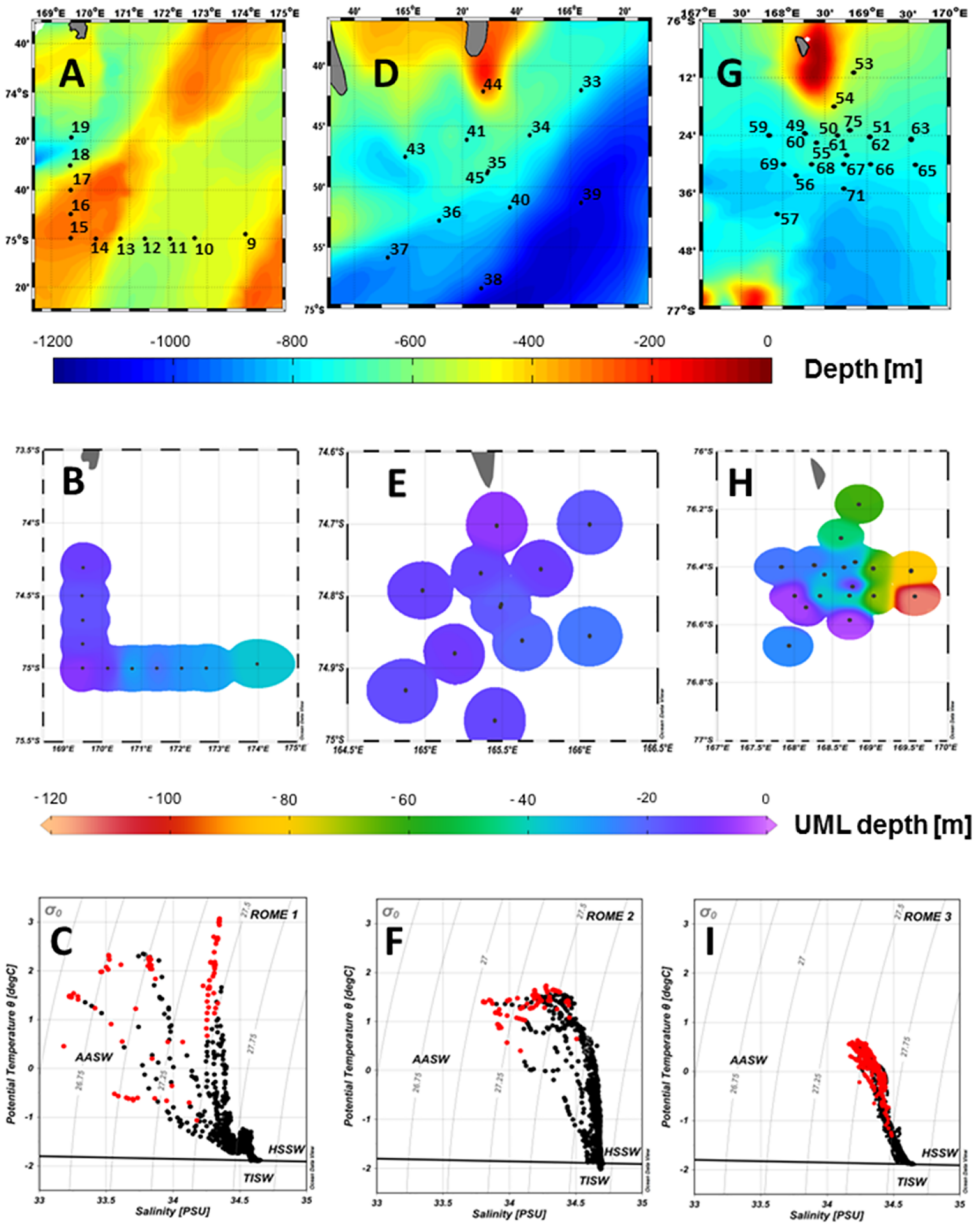
Sampling stations within each area, corresponding map of the upper mixed layer (UML) and the Θ/S diagram. Station maps for the RoME 1, 2 and 3 (A, D, G, respectively). The bathymetry data of the Ross Sea from [43] under a CC BY license, with permission from [Davey], original copyright [2004–2005]. Map of the UML depth (colour scale) for the three areas (B, E, H). The Θ/S diagrams for each leg with indication of data shallower than the UML (red dots) and of the freezing point (C, F, I). UML maps and Θ/S diagrams produced through Ocean Data View software [44].

The sampling strategy was designed on the basis of real-time satellite data, and was aimed at covering three areas that are characterised by different sea surface temperatures and chlorophyll-a (Chl-a) signatures, which appear to be related to the differences in water mass properties and dynamics. Maps from the Moderate Resolution Imaging Spectroradiometer Aqua and Terra satellite level-2 (products for the previous 12/24 h) were used to define the locations of the sampling stations. Sea ice conditions were obtained from the Advanced Microwave Scanning Radiometer-2 satellite maps provided by the University of Bremen (Germany). The sampling activity was then performed in three different areas of the Ross Sea (Fig 1; for list of geographical coordinates of stations see S1 Table). Fig 2 shows the sampling stations within each area, with a map of the upper mixed layer (UML) and Θ/S diagram.

First, the offshore sampling area was named RoME 1 (carried out from 16 to 17 January, 2014) and it was located in the northern section of the Ross Sea, between 74°S and 75°S, at approximately 170°E (Fig 2A). The “L” shape of this leg, which crossed Crary Bank, was designed to identify the location of the thermohaline fronts connected to expected differences in temperature and salinity between the more coastal and offshore water masses.

The second sampling area was named RoME 2 (carried out from 26 to 28 January, 2014) and it was located more coastward, next to the Terra Nova Bay polynya over the Drygalski Ice Tongue (Fig 2D). According to a clear signature in the satellite Chl-a maps and to the bathymetry forcing, it displayed a frontal structure in several oceanographic variables, oriented in the NE/SW direction [45].

Finally, the third sampling area was named RoME 3 (carried out from 31 January to 3 February, 2014), and it was situated in the offshore area of the southern Ross Sea at approximately 76.5°S, in a position south of Franklin Island and on the margin of the Ross Sea polynya (Fig 2G). This site was chosen due to the presence of an isolated mesoscale structure observed in the Chl-a concentration and the surface temperature satellite maps.

A total of 43 stations were sampled: RoME 1, 11 stations; RoME 2, 12 stations; and RoME 3, 20 stations (S1 Table). Continuous data and water samples were collected using a carousel sampler (Sea-Bird Electronics 32) equipped with 24 12-L Niskin bottles, a conductivity–temperature-depth (CTD) instrument (9/11 Plus; Sea-Bird Electronics) with double temperature and conductivity sensors, an oxygen sensor (Sea-Bird Electronics), a fluorometer (Chelsea Aquatracka III) and a programmable sonar altimeter (Datasonics). Furthermore, two lowered acoustic Doppler current profilers were deployed together with the CTD, to obtain current fields every 10 m from the surface to the maximum sampled depth. The water sampling depths (6–7 for each station) were chosen according to the fluorescence profile. Subsamples drawn from each Niskin bottle were collected for analysis of inorganic nutrients, particulate matter and phytoplankton (i.e., biomass, size classes, functional groups, taxonomic composition, photophysiology). Incident irradiance (LI-193SA quantum sensor; Licor) was measured during the whole length of the cruise.

### Hydrography, nutrients and particulate organic matter

Full resolution CTD data from the surface to the maximum sampled depth (always ≥200 m) were used to describe the main water masses and the water column characteristics during the three RoME legs. For each station, the UML depth was determined as the depth at which the *in-situ* density (*σt*) changed by 0.05 kg m^-3^ over a 5 m depth interval [45]. The melt-water percentage (MW%) was calculated from the difference between the salinity measured at a given depth (S_meas_) and the deep salinity (S_deep_; i.e., at 200 m), assuming a mean sea-ice salinity of 6 [46].

For determination of the nutrients (NO_3_^-^, NO_2_^-^, NH_4_^+^, Si(OH)_4_, PO_4_^3-^), samples were taken directly from the Niskin bottles, filtered through GF/F filters, stored at –30°C in 100 mL low-density polyethylene containers, and analysed using a five-channel continuous flow autoanalyser (Technicon Autoanalyser II), according to the method described by [47], which was adapted to the present instrumentation.

Particulate organic carbon (POC) and particulate organic nitrogen (PON) were analysed after acidification with HCl fumes, to remove inorganic carbon [48]. Cyclohexanone 2-4-dinitrophenyl hydrazone was used to calibrate an elemental analyser (Model 1110 CHN; Carlo Erba). The particulate protein and carbohydrate concentrations were determined [49, 50]. Albumin and glucose solutions were used to calibrate the spectrophotometer (Jasco V530).

### Phytoplankton pigments, taxonomic composition and photophysiology

Samples for determination of the total phytoplankton biomass and size structure (as micro [>20 μm], nano [2–20 μm], pico [<2 μm] fractions) were collected at 6 or 7 depths, following a protocol of serial filtration [51]. Filters were stored at -80°C until further analysis. The analyses of Chl-a and phaeopigments (Phaeo-a) were carried out according to [52], with a spectrofluorometer (Varian Eclipse), which was checked daily with a Chl-a standard solution (from *Anacystis nidulans*; Sigma).

For pigment spectra samples, 1 L or 2 L seawater was filtered (GF/F Whatman) and stored at -80°C for HPLC (1100 Series, Hewlett Packard) analyses [53]. Instrument calibration was carried out with external standard pigments provided by the International Agency for ^14^C determination-VKI Water Quality Institute. The relationship between spectrofluorimetric Chl-a and HPLC Chl-a for all samples was very close (p <0.001, y = 1.28 x + 0.26, R^2^ = 0.82, n = 198). The concentrations of pigments were used to estimate the contributions of the main functional groups to the total Chl-a using a matrix factorisation programme (CHEMTAX) [54, 55]. The carotenoids participating in the photoregulatory processes through the xanthophyll cycle were analysed [56, 57]. The photo-protective pigment ratio was calculated as the ratio of the sum of diadinoxanthin (Dd) and diatoxanthin (Dt) to Chl-a (Dd+Dt/Chl-a). The de-epoxidation state of the xanthophyll cycle was expressed as the ratio between the Dt and Dd+Dt concentrations (Dt/[Dd+Dt]).

Previous investigations on the interaction between Fe, diatoms and nutrient uptake highlighted a preferential drawdown of silicate under Fe-deplete compared to Fe-replete conditions, which resulted in an increase in the ratio between used silica and fucoxanthin [58]. In the present study, this ratio was computed as the difference between the mean Si(OH)_4_ concentration at 200 m in each RoME experiment and the corresponding Si(OH)_4_ concentration at a certain depth divided by the corresponding fucoxanthin concentration (ΔSi/Fuco). The ΔSi/Fuco ratio has been used as a proxy of Fe availability.

Samples for the phytoplankton taxonomic identification were collected at 4 or 5 depths, according to the vertical fluorescence profiles, and preserved in formalin solution (final concentration, 4%), buffered with CaCO_3_. Cell counts were performed with an inverted light microscope (Zeiss Axiophot), according to the Utermöhl method [59].

At each station (at 4–5 depths), aliquots of 30 mL water samples were rapidly collected from the Niskin bottles to measure the quantum yield. The photochemical efficiency of photosystem PSII was estimated by pulse amplitude fluorescence measurements using a fluorometer (Phyto-PAM; Walz, Effeltrich, Germany), with the PhytoWIN software used for the data elaboration [60]. After each water sample had been left in a dark-adapted environment for 10 min, 3 mL were injected into a quartz cuvette to determine the maximum quantum yield (F_v_/F_m_) of the photochemical energy conversion in PSII.

### Statistical analyses

Descriptive statistics (i.e., box plots, scattergrams, means, minima, maxima, standard deviations), correlation (Spearman) and multivariate (principal component analysis [PCA]) analyses were carried out using the XLSTAT software. PCA based on a correlation matrix was used to investigate the relationships among the *in-situ* environmental variables (temperature, melt-water fraction, nutrient concentrations) and biological features (F_v_/F_m_, ΔSi/Fuco, relative contribution of diatoms and Haptophytes) using the complete dataset (all of the selected parameters analysed in the three experiments).

## Results

### Physical and chemical constraints

During the entire sampling period, weather conditions were characterised by cloudiness, which partially influenced the satellite map availability and reduced the irradiance, with incident light ranging from 17 to 54 mol photons m^-2^ d^-1^. The means of the incident light during the RoME 1 and RoME 3 samplings were 30 and 28 mol photons m^-2^ d^-1^, respectively, while the RoME 2 sampling activities were characterised by higher daily irradiance levels, ranging from 27 up to 54 mol photons m^-2^ d^-1^ (on 28 January).

The ice retreat had occurred at different times over these three areas. In particular, the RoME 2 sampling area was free from ice from early December, whereas the northernmost station of RoME 1 and RoME 3 showed ice until early January and late December, respectively [61].

The lowered acoustic Doppler current profiler data and geostrophic velocities calculated from full-depth CTD data (not shown) confirmed that different satellite sea surface temperature and Chl-a patterns were also associated with particular water mass dynamics in each area. During RoME 1, the current data showed weak northward currents for the western stations, and stronger currents on the eastern side of Crary Bank. Here, a frontal structure was also evident, with large temperature and salinity gradients between the eastern and western parts of the leg, corresponding to stations 13 and 14. A thermohaline front was the main feature of the RoME 2 leg too, which was extensively described on the basis of current meter and CTD data [45]. Fresher and colder water masses were confined to the coastal area on the eastern part of the transect from station 33 to 37. Saltier and warmer waters were found in the eastern and deeper section of the leg, corresponding to the Drygalski Ice Tongue. The current meter and CTD data acquired during RoME 3 showed cyclonic circulation associated with the shoaling of temperature and salinity isolines at the centre of the mesoscale structure observed in the satellite data.

The Θ/S diagrams (Fig 2C, 2F and 2I) showed typical Ross Sea water masses as the Antarctic Surface Water (AASW), the High Salinity Shelf Water (HSSW) and the Terra Nova Bay Ice Shelf Water (TISW) [62, 63]. Nonetheless, significant differences in temperature and salinity properties were found mainly in the surface layer during the three legs. To highlight the variability of the UML water masses, this study is thus focused on the upper 200 m, while a complete analysis of the water mass properties along the entire water column can be found in [45, 61].

**Fig 3:**
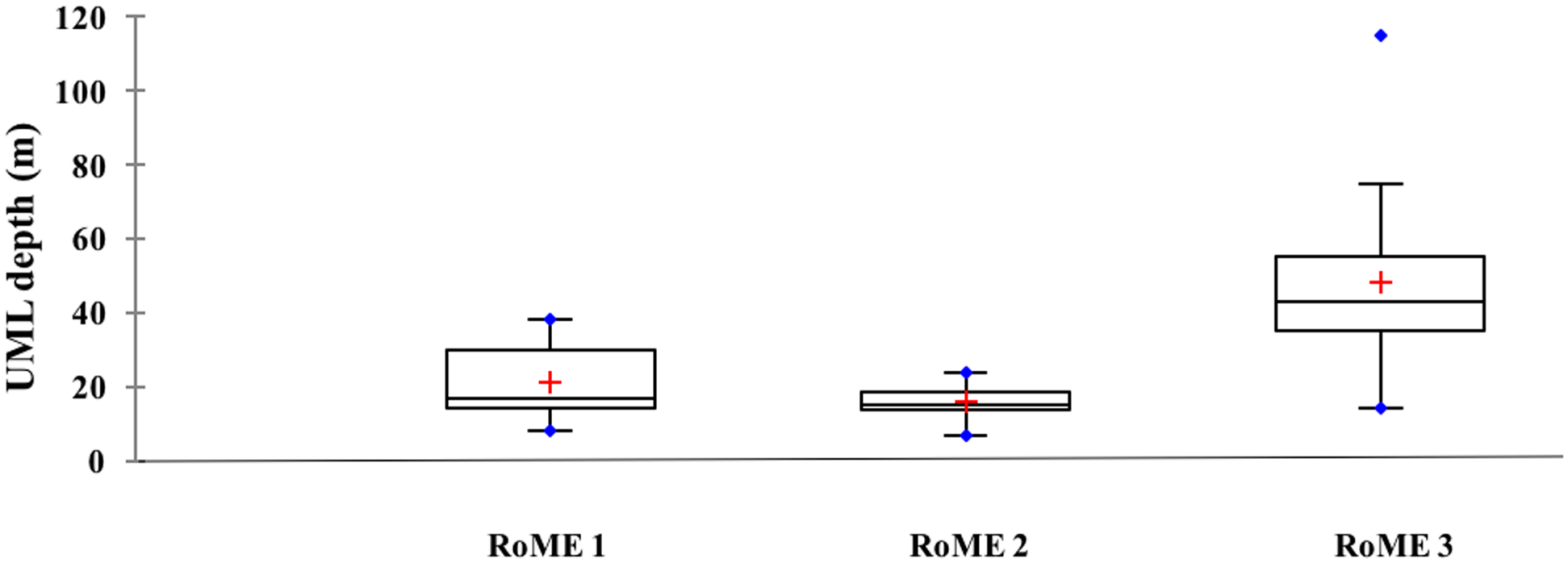
Upper mixed layer (UML) depth (m) in the three areas (boxplot by RoME). The red line shows the median, and the red cross the mean.

The intensity of the surface stratification is dependent on the freshwater input, melting sea-ice, and solar heating of the surface water. Consistent with the warmer temperatures and high melt water content at the surface, the water column was stratified, but differences in UML depth were observed (Figs 2B, 2E, 2H and 3). The mean thicknesses of the UML calculated for RoME 1 and RoME 2 were 21 m and 16 m, respectively. In contrast, the RoME 3 area was characterised by deeper UML and showed a mean that was about two-fold higher (48 m) compared to RoME 1 and RoME 2.

During RoME 1 the UML depth differed in space (Fig 2B) and was tightly coupled with the surface salinity distribution. For the eastern section (stations 9–13), which was characterised by longer ice-free conditions and separated from the low salinity coastal waters, the UML was deeper (23–38 m). On the other hand, stations 14 to 19 showed shallower mixed layers, which ranged between 8 m and 17 m. For RoME 2, the surface temperature and salinity showed low spatial variability, resulting in homogeneous UML depths, which ranged from 7 m to 24 m (Fig 2E). The RoME 3 was characterised by the presence of AASW and HSSW included in a cyclonic circulation, which was centred at about 168.5°E 76.45°S, and was associated to the strongest current intensities of the entire RoME cruise. This circulation pattern implied a shoaling of temperature, salinity and density isolines in the centre of the eddy and, as a consequence, enhanced the water mixing and increased the UML depth on the eddy border [64]. The shallowest UML depth was observed corresponding to the eddy centre, while a progressive deepening of the UML was observed at increasing distance from the centre (Fig 2H), especially in the north-eastern section of the area.

To examine the role of mixing dynamics in shaping the phytoplankton community, all of the data will be discussed by grouping the samples according to their depth. The first group includes all of the data at shallower depths than the UML, while the second group collects all of the data at deeper depths than the UML (Table 1).

**Table 1:** Mean (±standard deviations), minimum and maximum values, calculated in the upper mixed layer (UML) (A) and below the UML (B) for the three sampled areas.

	RoME 1						RoME 2						RoME 3					
	*Mean*		*Min*		*Max*		*Mean*		*Min*		*Max*		*Mean*		*Min*		*Max*	
	A	B	A	B	A	B	A	B	A	B	A	B	A	B	A	B	A	B
**Temperature**	1.75 (±1)	-1.41 (±0.3)	0.02	-1.86	3.07	-0.19	1.32(±1)	-0.86 (±1)	0.58	-1.91	3.54	1.54	0.35(±0.2)	-0.92 (±0.6)	0.10	-1.71	0.640	-0.01
**Salinity**	33.99 (±0.4)	34.44 (±0.1)	33.18	34.12	34.35	34.58	34.20 (±0.2)	34.62 (±0.1)	33.80	34.62	34.62	34.70	34.28 (±0.1)	34.45 (±0.1)	34.16	34.32	34.40	34.57
**DIN**	21.9 (±7)	30.4 (±7)	10.9	16.8	34.8	42.5	14.4 (±6)	27.5 (±5)	6.8	16.7	25.3	36.6	21.1 (±3)	28 (±4)	15.7	19.8	27.0	35.5
**PO**_**4**_^**3-**^	0.93 (±0.5)	1.61 (±0.2)	0.15	1.00	1.78	1.87	0.67 (±0.4)	1.49 (±0.3)	0.27	0.86	1.54	1.92	1.05 (±0.3)	1.67 (±0.4)	0.50	1.09	1.59	1.99
**Si(OH)**_**4**_	36.5 (±14)	61.6 (±16)	6.6	30.8	57.5	84.8	42.3 (±10)	66.5 (±15)	21.7	38.5	59.8	94.4	43.8 (±9)	58,5 (±17)	27.4	22	62.6	89
**POC PN**	274.4 (±89.7)	46.8 (±22.5)	139.3	23.3	444.2	93.7	208.3 (±23.4)	161.9 (±59.4)	166.4	52.1	233.5	256.0	227.9 (±84.4)	70.2 (±55.5)	45.4	24.6	350.7	198.7
**PON**	46.1 (±16.4)	6.6 (±3.3)	19.51	3.7	77.6	14.1	35.8 (±4.4)	22.6 (±8.9)	30.0	8.4	43.1	38.2	40.0 (±15.5)	11.6 (±10.9)	7.8	3.0	61.9	38.8
**Prot**	310.7 (±99.5)	53.0 (±26.6)	164.8	21.1	493.3	101.2	254.0 (±39.3)	162.8 (±72.0)	181.4	43.3	306.7	312.7	281.4 (±107.9)	87.2 (±81.2)	59.0	22.5	459.2	279.4
**Carb**	102.3 (±43.4)	21.7 (±8.3)	31.7	10.5	176.9	33.3	94.7 (±22.8)	98.2 (±54.7)	58.0	18.4	117.5	203.0	78.0 (±38.0)	20.2 (±13.8)	15.7	6.9	145.8	43.4
**POC/Chl-a**	209.2 (±119.8)	124.5 (±79.8)	84.0	51.7	495.3	317.1	161.8 (±(46.1)	66.9 (±8.6)	80.9	50.7	232.9	78.2	87.6 (±25.5)	109.7 (±60.3)	57.0	11.4	150.6	235.5
**POC/PON**	5.7 (±0.3)	6.6 (±1.0)	5.3	5.6	6.1	8.1	5.8 (±0.3	7.0 (±0.8)	5.4	5.9	6.3	8.9	5.8 (±0.5)	6.5 (±1.1)	5.1	5.1	7.5	8.9
**Prot/Carb**	3.3 (±0.8)	2.5 (±1.0)	2.4	1.3	5.2	4.5	2.8 (±0.5)	1.9 (±0.7)	2.3	1.1	3.6	3.4	3.8 (±0.8)	3.9 (±1.3)	2.5	1.8	5.6	6.5
**Chl-a**	1.50 (±0.4)	0.64 (±0.5)	0.56	0.07	2.37	1.66	1.86 (±0.8)	2.34 (±0.9)	0.58	0.71	3.54	3.79	2.95 (±1.3)	0.88 (±0.8)	0.51	0.10	4.71	3.06
**Phaeo/Chl-a**	1.15 (±0.8)	1.42 (1.2)	0.35	0.62	3.87	5.97	0.46 (±0.2)	0.45 (±0.1)	0.29	0.30	1.24	0.62	0.41 (±0.1)	1.04 (±0.9)	0.27	0.29	0.66	3.68
**ΔSi/Fuco**	49 (±30)	95 (±86)	24	0	140	314	91 (±37)	72 (±55)	40	0	184	196	38 (±20)	87 (±51)	18	6	112	165
**Diato**	77 (±15)	64 (±15)	50	27	89	84	67 (±16)	38 (±16)	33	15	98	74	93 (±2)	90 (±5)	90	65	99	97
**Hapto**	12 (±12)	26 (±13)	4	10	37	62	24 (±16)	54 (±16)	1	11	54	84	1 (±1)	3 (±3)	<0.1	0.5	3	11
**Micro**	68 (±14)	58 (±22)	37	15	87	93	73 (±11)	73 (±13)	51	43	86	85	82 (±12)	70 (±15)	51	50	96	92
**Nano**	22 (±11)	28 (±15)	7	13	52	68	15 (±9)	8 (±5)	4	0.4	38	22	12 (±12)	22 (±16)	2	3	45	48
**Pico**	12 (±7)	19 (±13)	2	7	23	45	12 (±4)	20 (±14)	7	6	23	48	7 (±3)	8 (±6)	2	2	14	16
**F**_**v**_**/F**_**m**_	0.25 (±0.1)	0.5 (±0.1)	0.15	0.37	0.44	0.65	0.34 (±0.1)	0.41 (±0.1)	0.19	0.22	0.49	0.60	0.58 (±0.1)	0.53 (±0.1)	0.22	0.40	0.64	0.66
**Dd+Dt/Ch-a**	0.24 (±0.06)	0.11 (±0.05)	0.12	0.06	0.40	0.24	0.11 (±0.06)	0.05 (±0.01)	0.04	0.02	0.24	0.07	0.14 (±0.04)	0.11 (±0.04)	0.1	0.06	0.25	0.23

Physical-chemical data: temperature (°C), salinity, inorganic nutrients [DIN ([NO_3_^-^ + NO_2_^-^ + NH_4_^+^), PO_4_^3-^, Si(OH)_4_] (μM) and POC [particulate organic carbon], PON [particulate organic nitrogen], Prot [protein] and Carb [carbohydrate] (mg m^-3^), POC/Chl-a, POC/PON, Prot/Carb. Biological and photophysiological variables: Chl-a (mg m^-3^), Phaeo/Chl-a [Phaeopigments/Chl-a], ΔSi/Fuco [ΔSi(OH)_4_/Fucoxanthin], Diato [diatoms] and Hapto [Haptophytes], micro (>20 μm), nano (2–20 μm) and pico (<2 μm) fractions (%), F_v_/F_m_ [maximum quantum yield], Dd+Dt/Ch-a.

**Fig 4:**
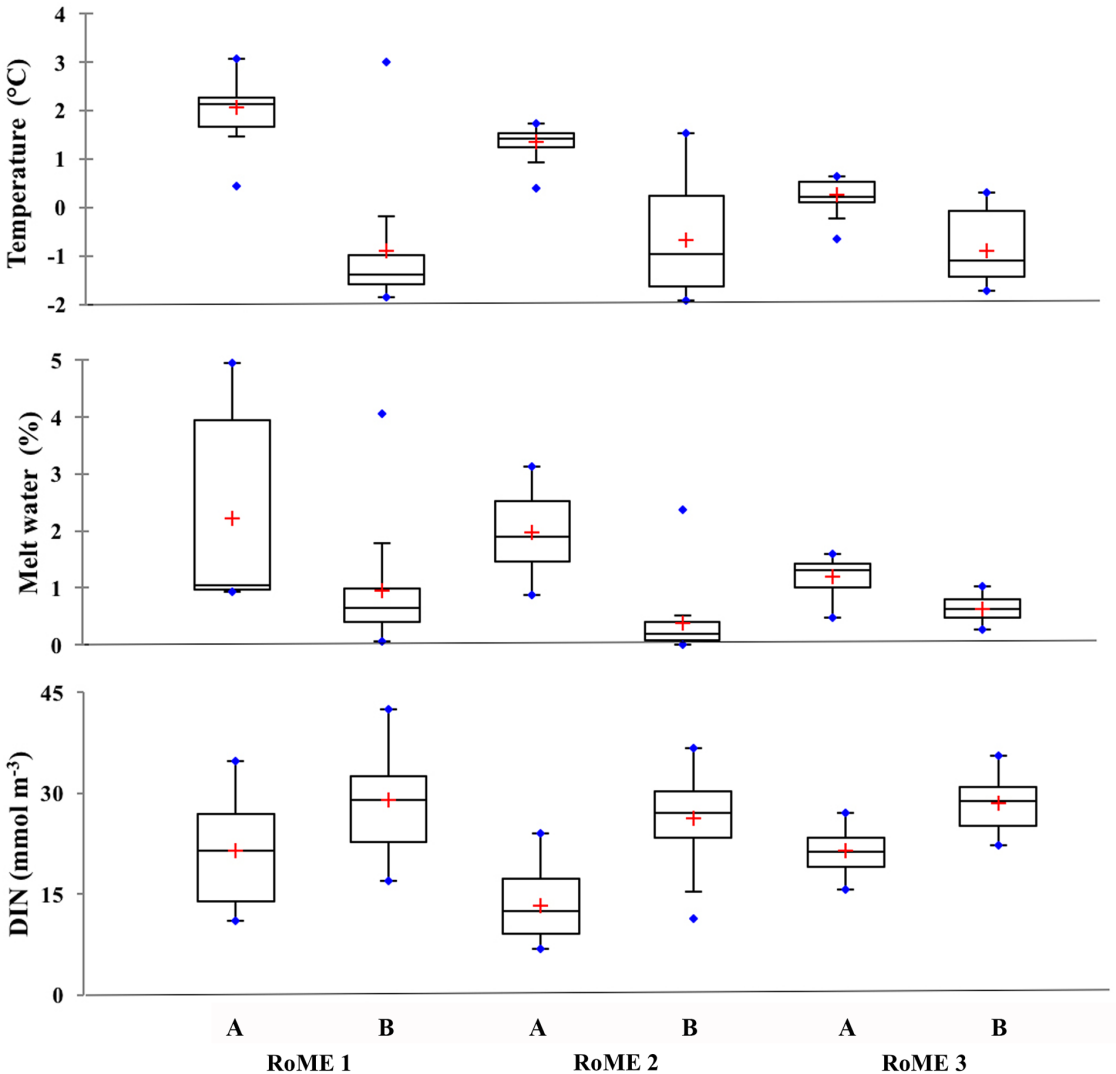
Environmental features in the two investigated layers of each area. Temperature (°C), melt-water (%) and DIN [dissolved inorganic nitrogen] concentrations (μM). The red cross shows the mean. Samples in the UML (A) and samples below the UML (B).

In the UML, the temperature was ≥0°C in almost all of the sampling stations (Fig 4). The highest temperatures (up to 3.07°C) were recorded in the easternmost stations of RoME 2, whereas the lowest temperatures were in RoME 3. Below the UML, the mean temperatures were similar in the three experiments, although RoME 2 showed the highest variability. The fraction of melt-water was highest (up to 4.96%) and mostly variable in the UML of RoME 1 (Fig 4). In the same layer, a progressive decrease of melt-water percentage was observed from RoME 2 (mean, 1.98%) to RoME 3 (1.20%). Below the UML, the percentage of melt-water decreased compared to the surface layers in all of the experiments. The lowest melt-water fraction (0.39%) was recorded during RoME 2. Dissolved inorganic nitrogen (DIN [NO_3_^-^ + NO_2_^-^ + NH_4_^+^]), PO_4_^3-^ and Si(OH)_4_ were never fully depleted in any of the investigated areas, and their concentrations were generally high and showed the lowest values at the surface, in the UML (Fig 4).

The POM concentrations, on average, were relatively similar for the three areas (Table 1), although slightly higher for RoME 3. Only the particulate carbohydrate concentrations were consistently higher in RoME 2 than in the other two areas. The POM concentrations decreased with depth, generally rather sharply for RoME 1 and RoME 3, with the highest concentrations observed in the UML. For RoME 2, this decrease was relatively smoother, with carbohydrate concentrations sometimes increasing in the 20 m to 50 m layer. The highest POC/PON ratios were in the deeper water layer (100 m), whereas the lowest ones (<6) were in the UML, especially for RoME 3, where the highest protein/carbohydrate ratios were also seen. The POC/Chl-a ratio was highest in RoME 1 (Table 1). Further details on the POM composition and distribution were reported by [61].

### Phytoplankton community structure and photophysiology

**Fig 5:**
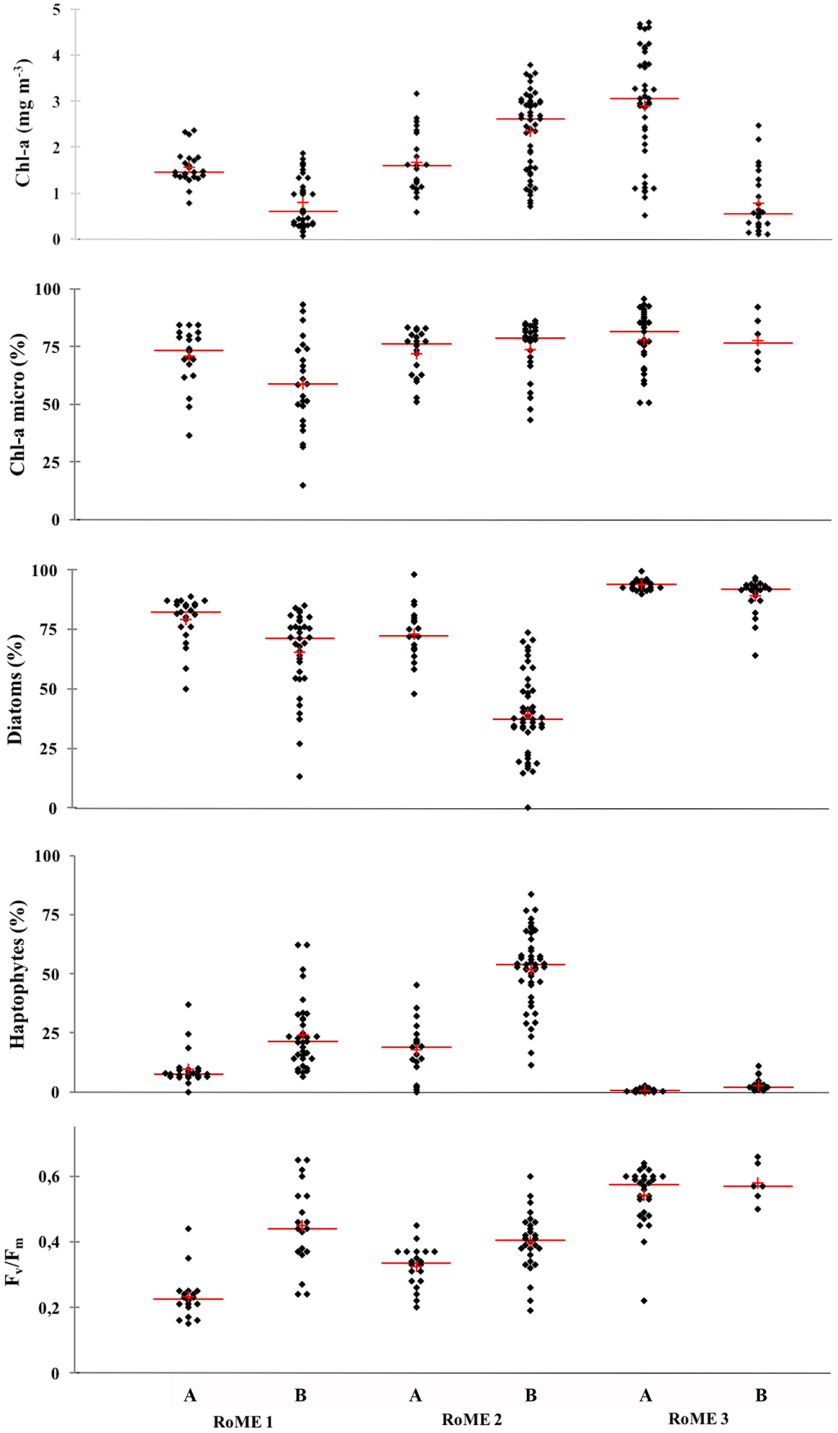
Biological features in the two investigated layers of each area. Total biomass (mg Chl-a m^-3^), micro (>20 μm) fractions (%), diatoms (%), Haptophytes (%) and F_v_/F_m_ [maximum quantum yield]. The red line is the median and red cross is the mean. Samples in the UML (A) and samples below the ULM (B).

The phytoplankton community and physiological characteristics will be discussed as related to the UML dynamics (Fig 5).

Stations were identified as diatom-dominated or Phaeocystis-dominated, based on CHEMTAX [65]. The most abundant pigments for all of the stations were fucoxanthin (mean concentration, 0.31 mg m^-3^) and 19-hexanoyloxyfucoxanthin (mean concentration, 0.16 mg m^-3^). All of the other accessory (non-chlorophyll) pigments (except diadinoxanthin, Dd) were present at <0.03 mg m^-3^.

In RoME 1, the integrated standing stock of Chl-a in the 0 m to 80 m layer varied from 44 mg m^-2^ to 110 mg m^-2^. At depths shallower than 20 m to 30 m (in the UML), the Chl-a concentrations ranged from 0.56 mg m^-3^ to 2.37 mg m^-3^, and decreased below the UML (0.07–1.66 mg m^-3^) (Fig 5 and Table 1). The analyses of the phytoplankton size fractions showed the dominance of micro-phytoplankton, mainly in the UML (means, 68% and 58%, in the UML and below the UML, respectively), whereas the contributions of the nano (20–2 μm) and pico (<20 μm) fractions were lower (Table 1). Phytoplankton were dominated by diatoms, with means of 77% in the UML and 64% below the UML. *Pseudo-nitzschia* spp. and *Fragilariopsis curta* were the most abundant diatom species, which varied from 50% to 90% and from 45% to 50%, respectively. The majority of the diatoms were found at their lower limits of cell size and in a senescent status. A small contribution of Haptophytes was observed, which was characterised by high variability and small increases (up to 62%) below the UML (Fig 5 and Table 1). In particular, the highest percentages of Haptophytes were recorded at the deepest layer of the northernmost coastal stations (17, 18, 19). In RoME 1, the F_v_/F_m_ measurements were low (mean, 0.25) in the UML, increasing up to a maximum (0.65) below the UML, at about 50 m depth (Fig 5). The vertical distribution of the photoprotective pigments, diadinoxanthin (Dd)+diatoxanthin (Dt) normalised to Chl-a (Dd+Dt/Chl-a), showed the highest values (0.12–0.40) in the UML, while the lowest values were recorded below the UML (0.06–0.24) (Table 1). The ratio of Dt to Dd was relatively higher below the UML.

In RoME 2, the integrated Chl-a concentrations varied from 133 mg m^-2^ to 371 mg m^-2^, which revealed standing stocks remarkably higher than RoME 1. The vertical profiles of Chl-a showed the presence of sub-surface maxima at 20 m to 40 m depth. The Chl-a concentrations in the UML ranged from 0.58 mg m^-3^ to 3.54 mg m^-3^ (mean, 1.86 mg m^-3^). Underneath the UML, Chl-a concentrations were highest at station 41 (mean, 2.34 mg m^-3^; reaching the highest value of 3.79 mg m^-3^), at 30 m depth. The micro-phytoplankton fraction (>20 μm) was the most abundant, which accounted for 73% of the total biomass in the entire water column (Fig 5). Phytoplankton community were co-dominated by both taxa. In the UML, the phytoplankton was mainly made up of diatoms, with means from 67% to the maximum of 98%. On the contrary, below the UML, the Haptophytes contribution increased to up to 54% of the total biomass. *Fragilariopsis* spp. and *Pseudo-nitzschia* spp. were the most abundant diatom species in the UML, whereas *Phaeocystis antarctica* in colonial forms dominated below the UML. In the stations where the diatoms were observed below the UML, they were all senescent, with empty frustules.

In the UML of RoME 2, the F_v_/F_m_ ratio was low (about 0.34), but slightly increased below the UML (mean, 0.41) (Fig 5). The (Dd+Dt)/Chl-a ratio was highest, but particularly variable (0.04–0.24) in the UML, and almost constant below the UML (about 0.05). The Dt/Dd ratio showed high variability, both in the UML and below the UML.

In RoME 3, the integrated Chl-a varied between 67 mg m^-2^ to 202 mg m^-2^ in the 0 m to 80 m layer, with the highest values occurring for the deepest UML. Chl-a concentrations ranged from 0.51 mg m^-3^ to 4.71 mg m^-3^ in the UML, and were generally <1.00 mg m^-3^ below the UML (Fig 5). In this area, diatoms were the almost exclusive taxa. Similar vertical profiles and low variability patterns were observed for all of the stations, characterised by a mean diatom percentage close to 90%, both in the UML and below the UML (Fig 5). The diatom species observed in RoME 3 were slightly different compared to the two other areas. As well as being dominated by *Pseudo-nitzschia* spp., *Dactlyliosolen* spp. was the other most abundant species. Most diatoms were in good condition, with cells dividing, many at their largest size and full of cytoplasm. In some stations, senescent diatoms with empty frustules were also observed, but only below the UML.

The F_v_/F_m_ showed high values at all depths, with a mean of 0.58. The Dd+Dt/Chl-a ratio was higher in the samples in the UML (0.14) compared to those below the UML (0.11). In addition, at the surface layer, the ratio did not show any appreciable variability and also the Dt/(Dt+Dd) ratio was relatively constant within the whole water column.

### Relationships between environmental and biological features

A multivariate approach was used to obtain information on the overall functioning of the three investigated areas. PCA based on correlations (Spearman) was used to investigate the relationships among the *in-situ* physical-chemical (temperature, melt-water percentage, nutrient concentrations) and biological (F_v_/F_m_, ΔSi/Fuco, relative contribution of diatoms and Haptophytes) variables.

**Fig 6:**
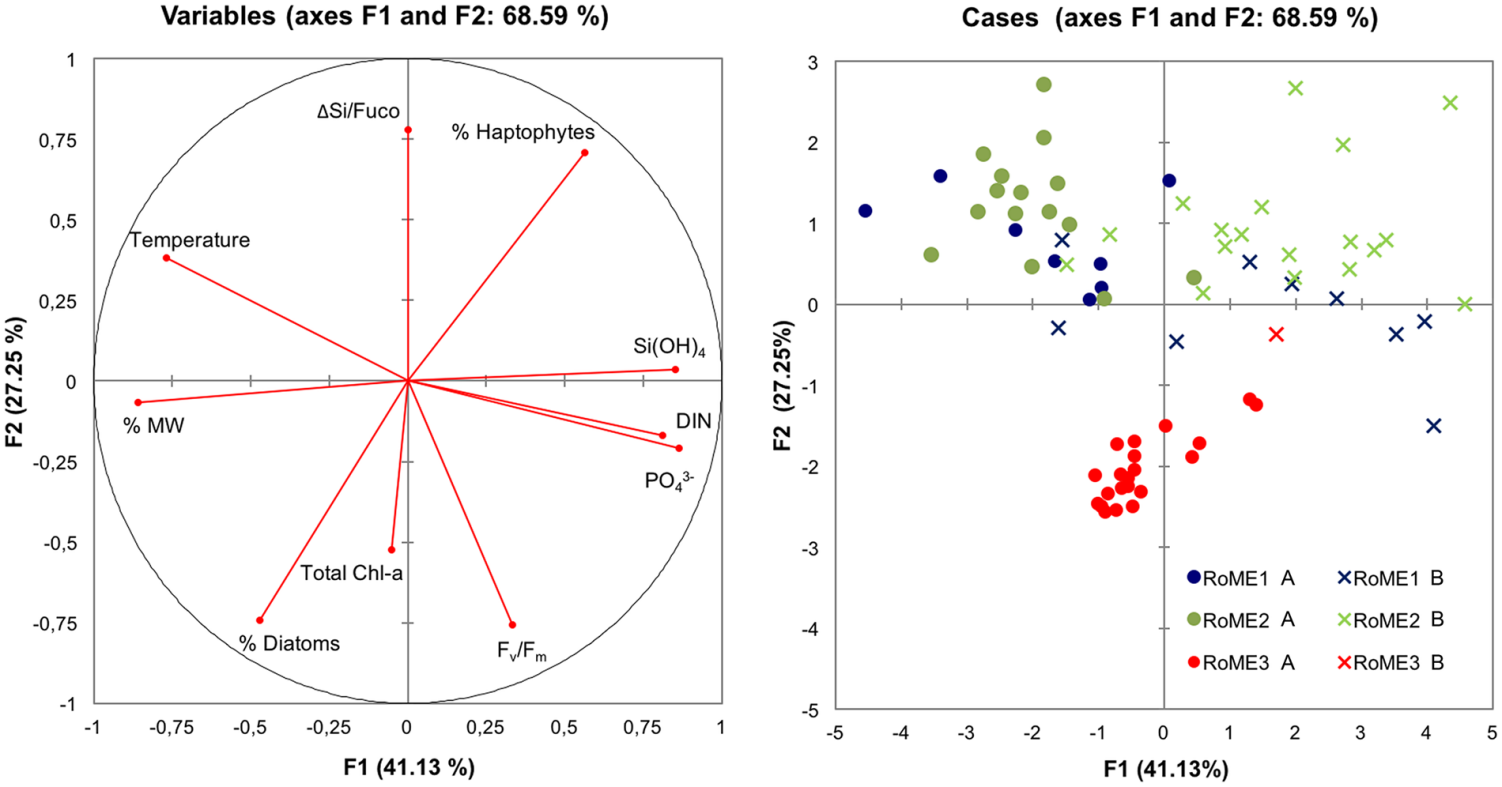
Results of PCA applied to physical, chemical and biological parameters. Parameters included in the analysis: ΔSi/Fuco [ΔSi(OH)_4_/Fucoxanthin], Haptophytes (%), Si(OH)_4_ [silicate], DIN [dissolved inorganic nitrogen (NO_3_^-^ + NO_2_^-^ + NH_4_^+^)], PO_4_^3-^ [phosphate], F_v_/F_m_ [maximum quantum yield], Total Chl-a, Diatoms (%), MW [melt-water] (%) and Temperature (°C). The samples in the UML (full shot-A) and samples below the ULM (cross-B): RoME 1, blu; RoME 2, green; and RoME 3, red.

The first two principal components (PCs), explained about 70% of the total variance, with the first accounting for 41%, and the second for 27% (Fig 6).

The first PC mainly explained the environmental variability. All the nutrient concentrations were negatively correlated to temperature and melt-water fraction, which thus indicated a dependence of nutrient concentrations from the sea-ice melting processes. The second axis explained the biological variability, where total Chl-a, F_v_/F_m_ and diatoms percentage were negatively correlated to the second axis, while the ΔSi/Fuco ratio (proxy got Fe availability) and the percentage of Haptophytes were positively correlated (Fig 6). The factor plane highlighted that the samples collected during the third experiment (RoME 3) were separated from samples collected during RoME 1 and RoME 2, and were mainly ordered following the biological variables. Samples collected during RoME 1 and 2 showed strong variability related to the physical and chemical variables, with different features mostly related to stratification (samples in the UML disposed in the fourth quarter and those below the UML in the first quarter of the ordination).

The percentage contributions of Haptophytes and diatoms to total biomass were negatively correlated. The diatoms positively correlated to the quantum yield and the melt-water percentage, and negatively to silicates concentration and to ΔSi/Fuco ratio, whereas opposite correlations were observed for Haptophytes (Table 2).

**Table 2:** Correlation matrix (Spearman) among physical, chemical and biological variables.

**Variables**	**F_v_/F_m_**	**DIN**	**PO_4_^3^**	**Si(OH)_4_**	**ΔSi/Fuco**	**TChl-a**	**Temp**	**MW**	**Hapto**	**Diato**
**F**_**v**_**/F**_**m**_	**1**	**0.296**	**0.370**	0.212	**-0.376**	**0.375**	**-0.605**	-0.218	**-0.305**	**0.354**
**DIN**	**0.296**	**1**	**0.810**	**0.617**	-0.135	-0.173	**-0.626**	**-0.579**	0.218	-0.187
**PO**_**4**_^**3-**^	**0.370**	**0.810**	**1**	**0.674**	-0.133	-0.100	**-0.669**	**-0.702**	**0.245**	-0.158
**Si(OH)**_**4**_	0.212	**0.617**	**0.674**	**1**	-0.091	-0.083	**-0.534**	**-0.715**	**0.492**	**-0.404**
**ΔSi/Fuco**	**-0.376**	-0.135	-0.133	-0.091	**1**	**-0.523**	0.180	-0.074	**0.453**	**-0.464**
**TChl-a**	**0.375**	-0.173	-0.100	-0.083	**-0.523**	**1**	-0.190	-0.004	-0.134	0.123
**Temp**	**-0.605**	**-0.626**	**-0.669**	**-0.534**	0.180	-0.190	**1**	**0.572**	-0.208	0.110
**MW**	-0.218	**-0.579**	**-0.702**	**-0.715**	-0.074	-0.004	**0.572**	**1**	**-0.516**	**0.404**
**Hapto**	**-0.305**	0.218	**0.245**	**0.492**	**0.453**	-0.134	-0.208	**-0.516**	**1**	**-0.920**
**Diato**	**0.354**	-0.187	-0.158	**-0.404**	**-0.464**	0.123	0.110	**0.404**	**-0.920**	**1**

Significant correlations (p <0.05) are shown in bold face. Variables: F_v_/F_m_ [maximum quantum yield], DIN [dissolved inorganic nitrogen (NO_3_^-^ + NO_2_^-^ + NH_4_^+^)], PO_4_^3-^ [phosphate], Si(OH)_4_ [silicate], ΔSi/Fuco [ΔSi(OH)_4_/Fucoxanthin], TChl-a [Total chlorophyll-a], Temp [temperature] MW [melt-water], Hapto [Haptophytes], Diato [diatoms].

Correlation analyses among the phytoplankton and POM quantity and composition were also carried out to achieve information on the trophic status of the investigated areas (Table 3). The results suggest a strong dependence of POM attributes on the main phytoplankton functional groups: diatoms were highly correlated with the quantitative features of POM, whereas Haptophytes were mostly correlated with the qualitative characteristics of POM.

**Table 3:** Spearman correlation matrixes between the phytoplankton and the POM features (quantitative: POC, protein and carbohydrate concentrations; qualitative: Protein to carbohydrate ratio, and POC/PON ratio) for the three sampled areas.

	**Variable**	Correlation with					
		**TChl-a**	**Phaeo**	**Micro**	**Pico**	**Hapto**	**Diato**
**RoME 1**	**POC**	***0*.*76***	**0.60**	**0.66**	-	-	***0*.*84***
	**PON**	***0*.*74***	**0.57**	**0.64**	-	-	***0*.*82***
	**Prot**	***0*.*78***	**0.59**	***0*.*70***	-	-	***0*.*86***
	**Carb**	***0*.*72***	0.53	**0.67**	-	-	***0*.*82***
	**Prot/Carb**	-	-	-	-	-	-
	**POC/PON**	**-0.61**	-0.45	-	-0.57	-	-0.60
		**TChl-a**	**Phaeo**	**Micro**	**Pico**	**Hapto**	**Diato**
**RoME 2**	**POC**	0.43	-	-	***-0*.*70***	-	**0.71**
	**PON**	-	-	-	***-0*.*80***	-	**0.60**
	**Prot**	-	-	-	***-0*.*69***	-	**0.70**
	**Carb**	***0*.*66***	**0.59**	**0.62**	-	-	0.66
	**Prot/Carb**	-0.52	**-0.62**	-	-	**-0.58**	-
	**POC/PON**	0.49	0.55	***0*.*69***	-0.57	***0*.*68***	-
		**TChl-a**	**Phaeo**	**Micro**	**Pico**	**Hapto**	**Diato**
**RoME 2**	**POC**	***0*.*92***	***0*.*86***	***0*.*76***	0.48	0.38	***0*.*95***
	**PON**	***0*.*92***	***0*.*85***	***0*.*77***	-	0.39	***0*.*94***
	**Prot**	***0*.*92***	***0*.*85***	***0*.*74***	-	0.38	***0*.*94***
	**Carb**	***0*.*88***	***0*.*84***	***0*.*78***	0.57	-	***0*.*93***
	**Prot/Carb**	-	-	-	-	-	-
	**POC/PON**	-	-	-	-	-	-0.43

Underlined numbers: p <0.05, bold numbers: p <0.01, italic-bold numbers: p <0.001, dash: not significant. POC [particulate organic carbon], PON [particulate organic nitrogen], Prot [proteins], Carb [carbohydrates], Prot/Carb, POC/PON, TChl-a [total chlorophyll-a], Phaeo [pheopigments], micro (>20 μm), nano (2–20 μm) and pico (<2 μm) fractions (%), Hapto [Haptophytes], Diato [diatoms].

## Discussion

### Ecological and photophysiological features of phytoplankton

During the austral summer of 2014, well-defined peculiarities in the qualitative and quantitative distributions of phytoplankton were observed in the three investigated areas of the Ross Sea, linked to water column dynamics and nutrients (i.e, availability, uptake, cycling). Physiological characteristics and xanthophyll pigments support the observed differences.

Two phytoplankton blooms occurred: the first was in the southernmost sampling stations (RoME 3), while the second was in the Terra Nova Bay polynya area (RoME 2). For RoME 1 (northern part of the Ross Sea), only a post-bloom phase was identified.

In the RoME 1 area, relatively low phytoplankton biomass was measured compared to the other two sub-systems. These results are comparable with other data collected during summer cruises in the Ross Sea in the 1990s, which were characterised by Chl-a concentrations that rarely exceeded 60 mg m^-2^ [66, 67]. Diatoms dominated in the RoME 1 area, as mostly inactive or dead cells. The high POC/Chl-a ratios observed reveal the low contributions of phytoplankton to particulate carbon. These findings are in agreement with the low values of the photosynthetic quantum yield, which indicates phytoplankton stress conditions, probably related to Fe limitation. The quantum yield of phytoplankton of the Southern Ocean is Fe-driven, and low values should be indicators of Fe stress [68]; the F_v_/F_m_ values below 0.40 suggest that Fe is the most relevant limiting factor [38, 69]. Several studies have reported an increase in the total pool of xanthophylls—diadinoxanthin (Dd) and diatoxanthin (Dt)—under stress conditions due to Fe deficiency [42] [70–72], even if the xanthophylls pool is traditionally used to couple the phytoplankton acclimations to light and water column dynamics [73, 74]. In the present study, the high values of (Dd+Dt)/Chl-a recorded in RoME 1 in the UML might be a combination of prolonged high light exposure, related to the high stability of the water column, and stress conditions, linked to the end of the bloom. Furthermore, the unusual high Dt/(Dd+Dt) ratio (Table 1) recorded below the UML in RoME 1 might be explained by the strong water column stratification. Recent studies focused on the Southern Ocean highlighted relatively high values of Dt/(Dd+Dt) observed below the mixed layer as result of chlororespiration, which was activated when phytoplankton were exposed to prolonged darkness [39, 75].

Different phytoplankton features were observed in RoME 2, coupled with the shallowest UML. The highest values were found for integrated biomass, which exceeded 300 mg Chl-a m^-2^, and exceptionally high concentrations were recorded below the UML (up to 3 mg m^-3^ at 100 m). These Chl-a concentrations were higher compared to those reported in the studies conducted before 2000, both in offshore and coastal areas of the Ross Sea [14, 21, 67, 76]. Recent studies focused on the Ross Sea have shown that during summer, phytoplankton biomass escapes from the ecological paradox of high-nutrient low-chlorophyll conditions. For instance, the prolonged ice seasons recorded in 2001 [25] and February 2004 [26] resulted in Chl-a concentrations greater than 6.0 mg m^-3^ in the southern Ross Sea. In 2011, Chl-a concentrations as high as 15 mg m^-3^ in the UML (about 50 m) were observed in the waters of the Pennell Bank [77, 78]. An intriguing feature for RoME 2 concerns the co-occurrence of diatoms and *P*. *antarctica*. The two investigated layers showed diverse phytoplankton community in different physiological status, characterised by diatoms in the UML and *P*. *antarctica* in the layer below the UML. The *P*. *antarctica* colonial bloom occurs in an area and in a season that are usually characterised by the prevalence of diatoms. Mucilage is known for its embedding floating of POM and DOM, to which the quantity of scavenged organic matter increases with the age of mucilage [79, 80]. The concentration of other POM components, such as proteins, was not significantly higher than in the other areas, which suggests that the production of mucilage by *P*. *antarctica* was recent. Samples in the UML showed the lower F_v_/F_m_ compared to below the UML. Thus, this suggests good adaptation of *P*. *antarctica* to low light conditions and nutrient availability, and an adequate amount of Fe, as reported by [71]. The levels of Fe detected [81] supports this hypothesis. Under Fe-replete regimes, compared to diatoms, *P*. *antarctica* is more able to efficiently use a large range of light levels, which are typical of a deep mixed layer [28, 82].

In the RoME 3 area, a second bloom was observed, which was confined in a wide UML (mean depth, 48 m) and characterised by biomass concentrations of up to 202 mg Chl-a m^-2^. In this area, diatoms represented more than 90% of the entire phytoplankton community. *Pseudo-nitzschia* spp. were the most abundant diatoms, and they were found at the higher limit of their cell size, which indicates that the phytoplankton were in an active growth phase. Moreover, the F_v_/F_m_ was the highest (mean, 0.55 ±0.09) and it was in the optimal range (0.45–0.65) reported for the Ross Sea [83], which suggests the onset of a phytoplankton bloom, not affected by limiting factors (such as Fe availability). During the study period, the uplift of the deeper waters that was favoured by the observed cyclonic circulation might have facilitated the episodic injection of deep Fe-rich waters into the euphotic zone [84]. The presence of recurrent mixing events can be also deduced by the low variability of photoprotective pigments within the UML.

An interesting finding of this study is the presence of large diatoms also in a wide UML, which contradicts the classic paradigm by which Antarctic diatoms generally accumulate in highly stratified waters. The high percentage of diatoms appears to be independent on the thickness of the UML (Figs 3 and 4) and the results of the PCA ordination stress that diatoms were positively correlated to melt-water percentages, whereas the opposite correlation was found for Haptophytes (Fig 6). This suggests that the melting processes might have a pivotal role in shaping the phytoplankton composition, more than the water column dynamics alone (at least in terms of the UML thickness). As climate change has a strong impact on the sea-ice dynamics at both global [85] and regional [86] scales, it might also have considerable effects on the ecology and biogeochemistry of the Ross Sea, altering the distribution of the two main functional groups. A recent study showed that high temperatures are correlated with high diatom abundance, and low temperatures match high *P*. *antarctica* [87], although it is not yet clear whether there is a causal mechanism behind this connection. Our study did not show any correlation between temperature and the relative contribution of *P*. *antarctica* and/or diatoms. Although correlation does not allow to infer cause-effect relationships, we hypothesise that rising ocean temperature, as expected from climate change predictions, might have a more relevant indirect effect on phytoplankton community structure through modulation of the ice dynamics, rather than only direct effects on cell physiology.

### Trophic implications

The composition of the phytoplankton communities determines the fate and pathway of carbon through the oceanic systems [16–19] [88]. Considerable increases in phytoplankton biomass and large size structure (micro-fraction accounting for 75% of the total biomass on average, independent of the functional groups) suggest that the Ross Sea in summer could now be extremely productive and might have a powerful impact on the trophic structure of the entire ecosystem (e.g., alterations in herbivore sizes, changes in pelagic-benthic coupling, presence of big mammals, change in penguin diet and others), as recently pointed out [89]. As the standing stock of phytoplankton was remarkably high in summer 2014, the question arises as to whether these organic materials would enter the classical Antarctic summer trophic chain or would have another fate. Before 2000, during summer, the offshore waters under ice-free conditions were characterised by low concentrations of phytoplankton biomass, which was characterised by a nano-size dominated community; these were coupled with a long trophic chain and an efficient microbial loop. Our results show that the phytoplankton features in summer 2014 were more similar to typical spring conditions, as characterised by the dominance of micro-phytoplankton fractions along the ice edge, which sustains the energy transfers through the Antarctic short trophic chain of ‘diatoms-euphausiids-whales’ [76] [90–92]. The low Pheao/Chl-a ratios and phaeophorbide concentrations (data not shown), as proxy of grazing activity [93, 94] and the presence of senescent (instead of grazed) phytoplankton cells, suggest the presence during summer 2014 of a relatively scarce trophic efficiency.

The significant correlations, with the quantitative and qualitative features of the POM, highlighted that phytoplankton drive the POM distribution and composition. In particular, diatoms had a major role in POM accumulation. This was mostly true in RoME 3, where the functional parameters of the phytoplankton community indicated active production, supported by favourable hydrodynamic conditions as well as micro- and macro-nutrient availability. This, however, allowed also a high qualitative value of POM. In this case, the bloom was at its early and intense phase, so consumers could not have started to feed on it.

RoME 1 was characterised by low photosynthetic efficiency with the presence of senescent cells, in agreement with the age of the bloom and the experienced long ice-free conditions. The high POC/Chl-a ratios and the moderate nutritional quality of POM agree with these features. A recent exploitation of this trophic resource by zooplankton was excluded, due to the low Phaeo/Chla-a ratios, but the senescent phytoplankton cells might host a microbial community that actively degrades POM in the absence of grazing activity. This explains the high protein content of POM, probably due to bacterial biomass.

In RoME 2, the low significance of the correlations suggests a decoupling between POM and phytoplankton. However, the low POC/Chl-a ratios in this area and the low grazing pressure still indicated a dominant role of phytoplankton. As previously mentioned, RoME 2 was characterised by a relevant presence of *P*. *antarctica*, which only partially contributes to the total quantity of POM, while it has a major role in determining the POM quality. Indeed, *P*. *antarctica* produces mucilage, which is composed of polysaccharides, with high carbohydrate content. The correlations with the qualitative features of POM (i.e., protein/carbohydrate ratio, POC/PON ratio) indicate low nutritional quality, and thus the summer *P*. *antarctica* production would be a moderate trophic supply [95], also for those grazers that can feed on mucilage aggregates [96]. Thus, the inefficient grazing on phytoplankton would be linked to the phytoplankton functional groups, composed of non-palatable or low nutritional value matter.

Our findings show an uncoupled increase in large diatoms and primary consumers (as revealed by the patterns of phaeopigments), independent of the phase of the bloom. On the other hand, the bloom decline appears to host an active microbial community, as revealed by the high protein contents of POM. This indicates that during the summer of 2014, the fate of primary production and carbon export could have been a lot different from those reported previously in the same area [67, 76, 97].

## Conclusions

The considerable biomass and large size of the phytoplankton observed, in agreement with the recent literature, suggest relevant alterations in Ross Sea summer productivity. Moreover, the distribution of the main functional groups showed significant anomalies. The *P*. *antarctica* colonial bloom occurs in an area and in a season that are usually characterised by the prevalence of diatoms. The presence of large diatoms in a wide UML contradicts the classic paradigm of Antarctic diatom accumulation in highly stratified waters.

The imbalance between phytoplankton standing stocks and primary consumers, independent of the phase of the bloom, might dramatically alter the fate of the summer primary production and the carbon export in the Ross Sea. However, it is unclear what are the environmental factors that drive these extraordinary changes in primary production processes and the prevalence of different functional groups. Probably, a modification of Ross Sea hydrography will have a key role in reducing the limiting factors, and as consequence, in modifying the primary production processes.

Certainly, this present asset will affect not only the Ross Sea, but probably the entire Southern Ocean ecology, and subsequently it may have an impact at a global scale.

## Supporting information

S1 Table Sampling stations within each area of the RoME Project, corresponding geographic coordinates, bottom depth and sampling date. (DOCX)
