# Supplementary material for: Phytoplankton blooms during austral summer in the Ross Sea, Antarctica: Driving factors and trophic implications
Source: PLoS One. 2017 Apr 21;12(4):e0176033. doi: 10.1371/journal.pone.0176033 (PMC5400245; doi:10.1371/journal.pone.0176033)
Supplement: S1 Table — (DOCX) [file pone.0176033.s001.docx]

**S1 Table. Sampling stations within each area of the RoME Project, corresponding geographic coordinates, bottom depth and sampling date.**

| **Area** | **Station** | **Longitude**  **[degrees_east]** | **Latitude**  **[degrees_north]** | **Bottom depth [m]** | **Date** |
| --- | --- | --- | --- | --- | --- |
| RoME 1 | 9 | 173.874 | -75.001 | 454 | 16 Jan 2014 |
|  | 10 | 172.667 | -74.998 | 526 | 16 Jan 2014 |
|  | 11 | 172.035 | -75.001 | 545 | 16 Jan 2014 |
|  | 12 | 171.396 | -75.000 | 536 | 16 Jan 2014 |
|  | 13 | 170.763 | -75.003 | 464 | 16 Jan 2014 |
|  | 14 | 170.137 | -75.002 | 338 | 17 Jan 2014 |
|  | 15 | 169.496 | -74.999 | 326 | 17 Jan 2014 |
|  | 16 | 169.496 | -74.834 | 313 | 17 Jan 2014 |
|  | 17 | 169.499 | -74.670 | 346 | 17 Jan 2014 |
|  | 18 | 169.505 | -74.505 | 567 | 17 Jan 2014 |
|  | 19 | 169.498 | -74.331 | 722 | 17 Jan 2014 |
| RoME 2 | 33 | 166.065 | -74.701 | 705 | 26 Jan 2014 |
|  | 34 | 165.747 | -74.763 | 719 | 26 Jan 2014 |
|  | 35 | 165.488 | -74.813 | 674 | 26 Jan 2014 |
|  | 36 | 165.183 | -74.882 | 819 | 27 Jan 2014 |
|  | 37 | 164.870 | -74.932 | 890 | 27 Jan 2014 |
|  | 38 | 165.459 | -74.974 | 1035 | 27 Jan 2014 |
|  | 39 | 166.064 | -74.856 | 1080 | 27 Jan 2014 |
|  | 40 | 165.626 | -74.862 | 792 | 27 Jan 2014 |
|  | 41 | 165.357 | -74.768 | 699 | 27 Jan 2014 |
|  | 43 | 164.981 | -74.793 | 775 | 27 Jan 2014 |
|  | 44 | 165.469 | -74.703 | 189 | 27 Jan 2014 |
|  | 45 | 165.485 | -74.816 | 691 | 27 Jan 2014 |
| RoME 3 | 49 | 168.264 | -76.395 | 686 | 31 Jan 2014 |
|  | 50 | 168.651 | -76.401 | 659 | 31 Jan 2014 |
|  | 51 | 169.035 | -76.407 | 670 | 1 Feb 2014 |
|  | 53 | 168.850 | -76.185 | 512 | 1 Feb 2014 |
|  | 54 | 168.613 | -76.300 | 436 | 1 Feb 2014 |
|  | 55 | 168.397 | -76.427 | 676 | 1 Feb 2014 |
|  | 56 | 168.156 | -76.540 | 763 | 1 Feb 2014 |
|  | 57 | 167.933 | -76.674 | 781 | 1 Feb 2014 |
|  | 59 | 167.839 | -76.400 | 204 | 2 Feb 2014 |
|  | 60 | 168.268 | -76.394 | 202 | 2 Feb 2014 |
|  | 61 | 168.655 | -76.401 | 203 | 2 Feb 2014 |
|  | 62 | 169.034 | -76.404 | 203 | 2 Feb 2014 |
|  | 63 | 169.529 | -76.412 | 204 | 2 Feb 2014 |
|  | 65 | 169.576 | -76.502 | 726 | 2 Feb 2014 |
|  | 66 | 169.042 | -76.500 | 728 | 2 Feb 2014 |
|  | 67 | 168.721 | -76.499 | 711 | 2 Feb 2014 |
|  | 68 | 168.340 | -76.500 | 695 | 2 Feb 2014 |
|  | 69 | 168.009 | -76.500 | 756 | 2 Feb 2014 |
|  | 71 | 168.727 | -76.584 | 201 | 2 Feb 2014 |
|  | 75 | 168.796 | -76.384 | 645 | 3 Feb 2014 |
